# Network Pharmacology-Based Prediction of Catalpol and Mechanisms against Stroke

**DOI:** 10.1155/2021/2541316

**Published:** 2021-01-07

**Authors:** Jinghui Wang, Meifeng Zhang, Si Sun, Guoran Wan, Dong Wan, Shan Feng, Huifeng Zhu

**Affiliations:** ^1^College of Pharmaceutical Sciences &; College of Chinese Medicine, Southwest University, Chongqing 400715, China; ^2^Department of Clinical Medicine, Chongqing Medical University, Chongqing 400016, China; ^3^Department of Emergency and Critical Care Medicine, The First Affiliated Hospital of Chongqing Medical University, Chongqing 400016, China

## Abstract

**Aim:**

To apply the network pharmacology method to screen the target of catalpol prevention and treatment of stroke, and explore the pharmacological mechanism of Catalpol prevention and treatment of stroke.

**Methods:**

PharmMapper, GeneCards, DAVID, and other databases were used to find key targets. We selected hub protein and catalpol which were screened for molecular docking verification. Based on the results of molecular docking, the ITC was used to determine the binding coefficient between the highest scoring protein and catalpol. The GEO database and ROC curve were used to evaluate the correlation between key targets.

**Results:**

27 key targets were obtained by mapping the predicted catalpol-related targets to the disease. Hub genes (ALB, CASP3, MAPK1 (14), MMP9, ACE, KDR, etc.) were obtained in the key target PPI network. The results of KEGG enrichment analysis showed that its signal pathway was involved in angiogenic remodeling such as VEGF, neurotrophic factors, and inflammation. The results of molecular docking showed that ACE had the highest docking score. Therefore, the ITC was used for the titration of ACE and catalpol. The results showed that catalpol had a strong binding force with ACE.

**Conclusion:**

Network pharmacology combined with molecular docking predicts key genes, proteins, and signaling pathways for catalpol in treating stroke. The strong binding force between catalpol and ACE was obtained by using ITC, and the results of molecular docking were verified to lay the foundation for further research on the effect of catalpol on ACE. ROC results showed that the AUC values of the key targets are all >0.5. This article uses network pharmacology to provide a reference for a more in-depth study of catalpol's mechanism and experimental design.

## 1. Introduction

Stroke has a high prevalence, recurrence rate, disability rate, and mortality rate. But at present, there is a shortage of drugs in the clinical treatment of stroke [1]. Catalpol is one of the main active components of *Rehmannia glutinosa*, a traditional Chinese medicine for nourishing yin and kidney, especially the content of Radix Rehmanniae [2]. Although more researches showed that catalpol is a potential drug to treat senile neurodegenerative diseases such as cerebral ischemia [3, 4], diabetic encephalopathy [5], hyperlipidemia, and dementia, however, the target and mechanism of catalpol action are not clear, and further research and exploration are still needed.

The concept of network pharmacology was first put forward by British pharmacologist Hopkins in 2007 [6]. Based on the multidisciplinary theories of systems biology and multidirectional pharmacology, the molecular mechanism of drug intervention in diseases was understood from a multidimensional perspective. In order to further explore the target and mechanism of catalpol in the prevention and treatment of stroke, this paper is based on the systems biology and computer technology of “disease-gene-target-drug” interaction network. Firstly, we identified the potential targets of catalpol in the treatment of stroke and predicted the pathways and networks of catalpol and its effects.

**Figure 1:**
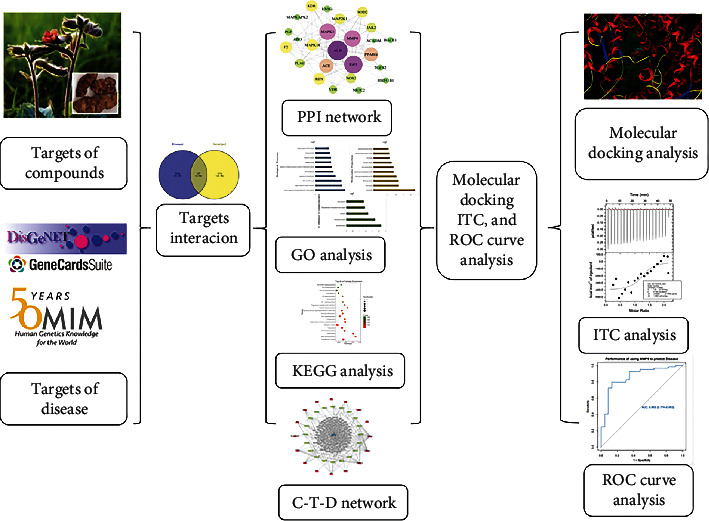
Workflow of validation and exploration of pharmacological mechanism of catalpol for stroke prevention and treatment based on network pharmacology.

In this study, molecular docking, isothermal titration calorimetry (ITC), and receiver operator characteristic (ROC) curve were added to network pharmacology as a supplement to predict drug targets. Molecular docking technology is based on the “lock and key principle” of ligand-receptor interaction and uses computer-aided drug design to conduct virtual drug screening, which is a quick and effective way to identify drug targets [7]. Isothermal titration calorimetry (ITC) is a technique that can directly measure the binding energy of biological processes, including protein-ligand binding, protein-protein binding, DNA-protein binding, protein-carbohydrate binding, protein-lipid binding, and antigen-antibody binding [8]. Here, we studied the docking of key hub protein to predict the interaction between catalpol and its predicted target, and then ITC was used to determine the binding coefficient of catalpol to the protein with the highest molecular docking score and to verify the results of molecular docking, based on the accurate determination of the heat released or absorbed during the formation of the complex. GEO (gene expression omnibus) database is based on gene chip technology and can be used to construct disease validation sets. After construction, the ROC curve can be used to evaluate the correlation between the key target and the disease. The workflow is shown in Figure 1.

## 2. Materials and Methods

### 2.1. Drugs

**Figure 2:**
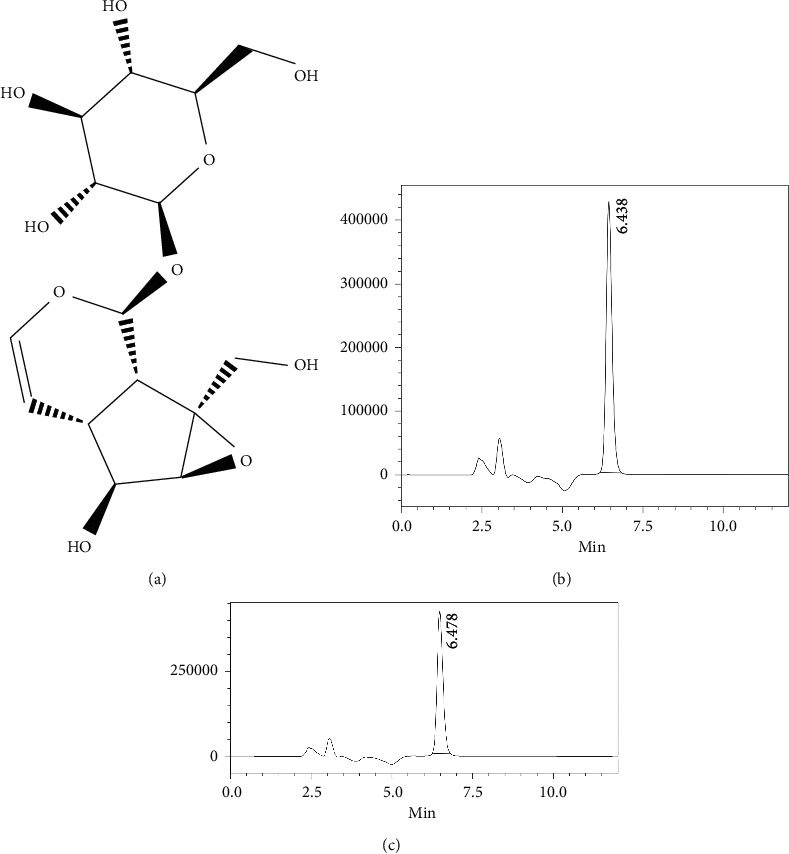
(a) Chemical structure of catalpol from the PubChem database (CAS: 219-324-0); (b) chromatogram of catalpol; (c) system suitability test of HPLC in determining the catalpol.

Catalpol (purity >98%, basic information of catalpol, see Figure 2) was purchased from Liubobainiao Biotechnology Co., Ltd. (Shijiazhuang, China). The enzymes ACE (3.7 U/mg protein) from rabbit lung were supplied by Sigma Chemical Co. (St. Louis, MO, USA).

### 2.2. Acquisition of Catalpol Target

The structure file of catalpol was obtained by logging into PubChem (https://pubchem.ncbi.nlm.nih.gov/), and the target was predicted by reverse pharmacophore matching PharmMapper database (http://lilab-ecust.cn/pharmmapper/) based on a pharmacophore model. The target name and the corresponding score are obtained, and the higher the score, the more the molecule and the target point match. The target protein of the compound was selected as the target protein of the compound, and the target protein database of the catalpol was established by using the UniProt database. Get the target name and the fit score, the higher the score, the more the matching. A score of >3 was selected as the target protein of the compound, and the UniProt database (http://www.uniprot.org/) was used for correction to establish a target protein database of catalpol.

### 2.3. Access to Stroke Disease Targets and Key Targets

Access to OMIM (https://omim.org/), DisGeNet (http://www.disgenet.org/), and GeneCards (https://genecards.weizmann.ac.il/v3/) databases with the key words “stroke” and “cerebral ischemia” search for stroke-related disease targets, summary, and removal of duplicate genes. Matching the target of the disease with the target of catalpol to obtain a common target is a potential target for catalpol treatment of stroke, and the key targets are obtained as further research. Key targets are imported into the DisGeNET database for target protein class.

### 2.4. Construction of PPI Interaction Network

Key targets are imported into the STRING database (https://string-db.org/cgi/input.pl), the species is defined as human, and the PPI interaction network is obtained. The result is saved in TSV format, the node1 and node2 information in the file is retained and imported into Cytoscape3.7.1 to draw the interaction network diagram. The size of degree is reflected by the node size and color settings, and the hub protein is obtained with the top 10 values in the PPI network. The target protein interaction network map is composed of multiple nodes connected to each other, and the hub node is more closely and importantly connected than other nodes.

### 2.5. Gene Function and Pathway Enrichment Analysis

DAVID (https://david.ncifcrf.gov/conversion.jsp) is a database for enrichment and analysis of genes or proteins, which can provide comprehensive and systematic information on biological function. Key targets are imported into the DAVID database, the species is defined as human, GO and KEGG analysis is performed on key targets, and the results are saved. They are sort by *P* value and the top biological processes or pathways are filtered for mapping. To understand the complex relationship between compounds, targets, pathway, and diseases, we use Cytoscape3.7.1 to build a network diagram.

### 2.6. Molecular Docking

Molecular docking is used to verify the binding activity between target proteins and active components. The protein structure of the key target was obtained from the RCSB PDB database (https://www.rcsb.org/), and the 3D structure of catalpol was obtained from the PubChem database (https://pubchem.ncbi.nlm.nih.gov/). SYBYL-X2.1.1 was used to hydrogenate the protein, find active pockets, and conduct molecular docking verification with catalpol. The total-score function of Surflex-Dock molecular docking module was used to score the interaction between active components and target proteins. The higher the total-score value, the better the matching binding between small molecular compounds and macromolecular proteins.

### 2.7. Isothermal Titration Calorimetry Binding Assays

Assays were performed on a ITC200 instrument (MicroCal, Northampton, MA). The experimental titration was carried out in the boric acid buffer system containing 0.3 M NaCl pH 8.3, and the ACE of the 75–125 nm of the buffer was dissolved in the hot pool. The temperature of the sample cell was set at 25∼30°C, the total titration times were 20, and the interval time was 60 s. Catalpol was titrated after the automatic balance of the calorimeter was completed. The thermodynamic parameters of the reaction (ΔG, ΔH, and ΔS) and dissociation constant K_d_ were calculated using Origin 7.0 with iTC200 MicroCal software.

### 2.8. Application of ROC Curve to Evaluate Key Targets

Using the relevant dataset in the gene expression omnibus (GEO), the receiver operator characteristic (ROC) curve was used to evaluate the correlation between the key target and stroke disease.

## 3. Results

### 3.1. The Action Target of Catalpol

In the PharmMapper database, the target protein of fit score >3 was selected as the target protein of catalpol, and the obtained protein target was input by UniProt database. By retrieval and transformation, the 201 potential targets of catalpol were obtained.

### 3.2. Screening of the Target of Disease and the Attribution of the Key Target

**Figure 3:**
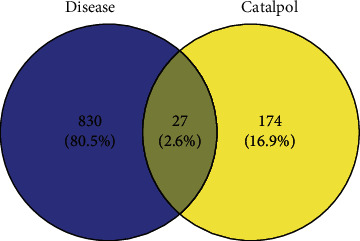
Matching map of disease target gene and catalpol target gene.

In OMIM, DisGeNet, GeneCards database retrieved 857 relevant disease target genes. Matching 857 disease target with catalpol active ingredient-related targets (3.1), 27 key targets were obtained (Figure 3). Protein class of key targets in the DisGeNET database is shown in Table 1. These targets belong to hydrolase, protease, nucleic acid binding, receptor, transcription factor, signaling molecule, transferase, carrier protein, etc.

### 3.3. Construction of PPI Protein Interaction Network

**Figure 4:**
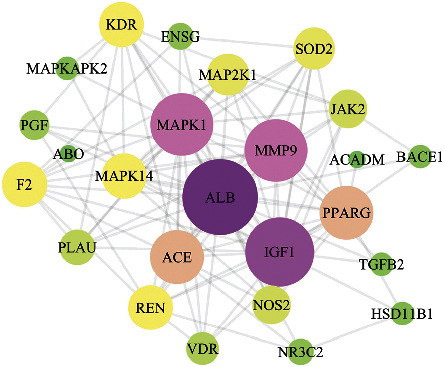
Key target PPI protein interaction diagram.

The abovementioned key target proteins are imported into the STRING database, the species is defined as human, the protein interaction relationship is obtained, the TSV format file is saved, and the Cytoscape3.7.1 is imported to draw the interaction network (Figure 4). The nodes in the figure represent the target, and the edges represent the associations between the targets. The size and color of the nodes represent the size of the degree. The larger the node, the larger the value of the color corresponding to the color from purple to green will be.

### 3.4. GO Biological Function and KEGG Pathway Enrichment

**Figure 5:**
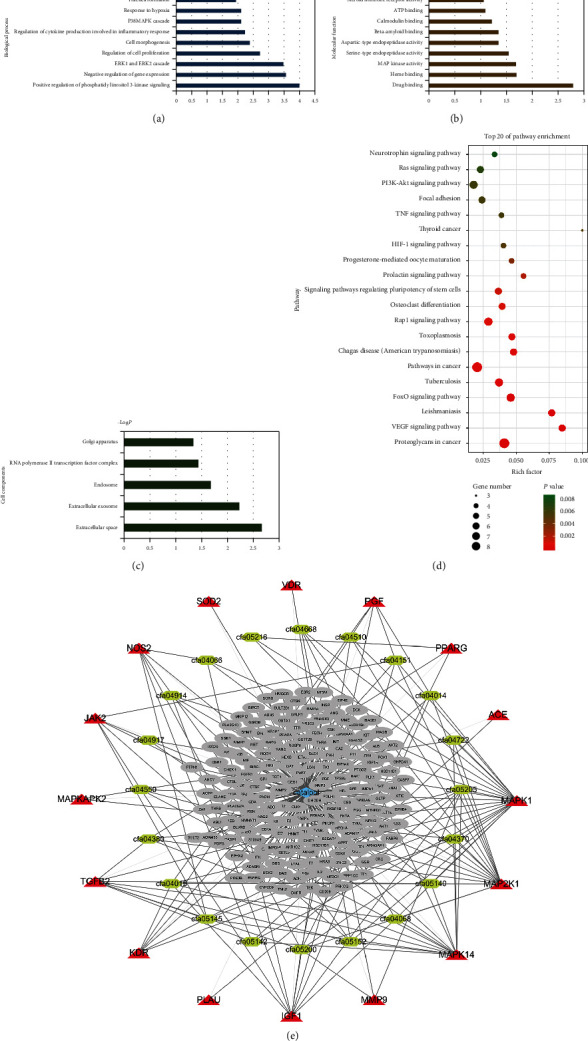
(a) Main potential targets for the treatment of stroke biological processes (blue); (b) cellular components (green); (c) molecular function (brown); (d) enrichment bubble map of KEGG pathway in the treatment of stroke with main potential targets of catalpol; (e) catalpol-key target-pathway network.

The results of GO enrichment analysis showed that catalpol was related to biological process (BP), cell component (CC), and molecular function (MF) (Figures 5(a)–5(c)). The KEGG enrichment analysis of the key targets was carried out, and the distribution results ranked in the top 20 were obtained (Figure 5(d); Table 2). KEGG enrichment analysis showed that the enrichment pathway was mainly related to nerve remodeling, inflammation, cancer, and parasitic diseases. MAPK signal plays an important role in the mechanism of catalpol in the prevention and treatment of these diseases. In order to show the relationship between catalpol, core target, and pathway more clearly, the catalpol-core target-pathway network was constructed by using Cytoscape3.7.1 software (Figure 5(e)). Blue catalpol, grey catalpol target, green signal pathway, and red represent the key shared node of catalpol signal pathway.

### 3.5. Molecular Docking

**Figure 6:**
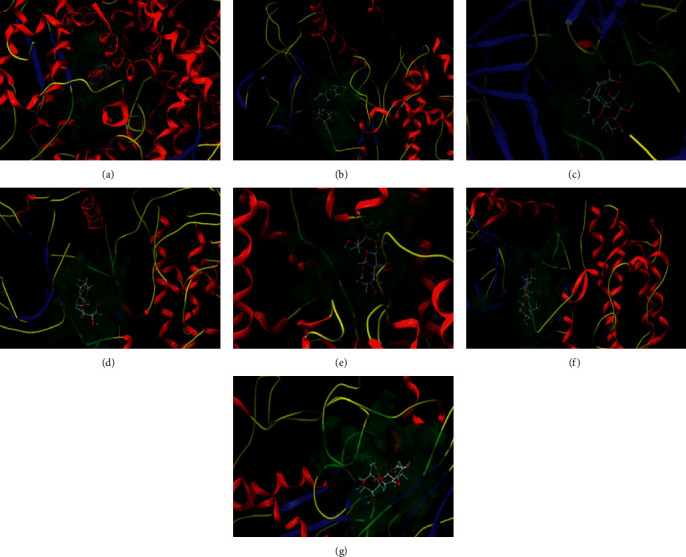
The interaction mode between catalpol and related proteins obtained by molecular docking technique. (a) ACE, (b) MAPK14, (c) REN, (d) MAPK1, (e) ALB, (f) KDR, and (g) F2.

In order to better explain the binding activity between hub protein and catalpol, molecular docking was carried out by SYBYL-X2.1.1 software, and the docking score is shown in Table 3. The docking score ≥4.25 indicates that there is a certain binding activity between the molecule and the target, the docking score more than 5.0 indicates a good binding activity, and the docking score more than 7.0 indicates that the molecule and the target have strong binding activity. The docking scores of ACE, MAPK14, REN, MAPK1, ALB, KDR, and MMP9 were ≥4.25, which showed good binding activity. The docking diagram is shown in Figure 6.

### 3.6. ITC Assays

**Figure 7:**
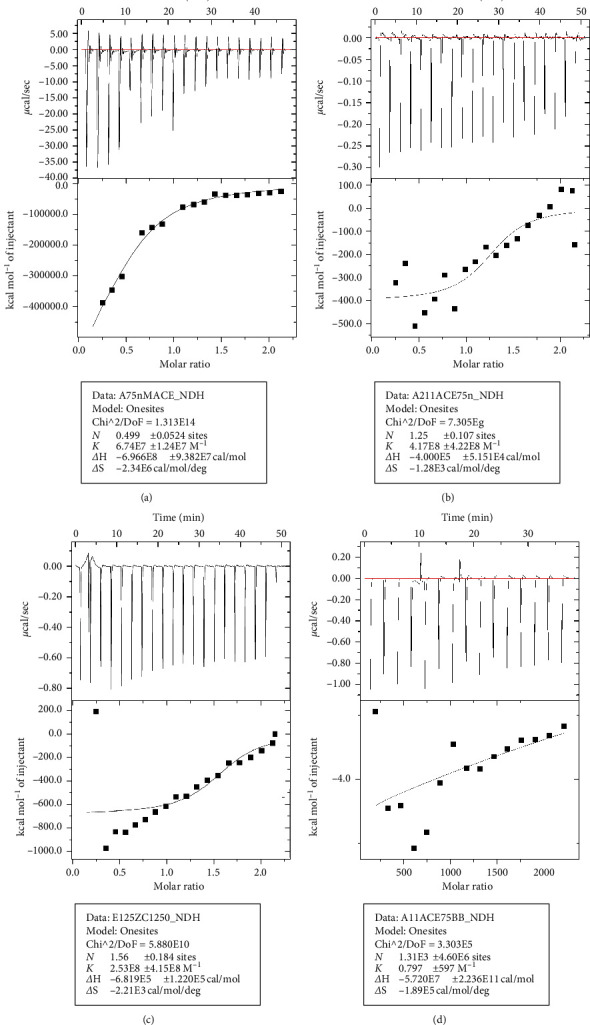
(a) 750 nm catalpol titration 75nMACE, temperature 25°C; (b) 750 nm catalpol titration 75nMAE, temperature 30°C; (c) 1250 nm catalpol titration 125nMAE, temperature 30°C; (d) boric acid buffer titration 75nMACE, temperature 30°C.

According to the results of molecular docking, the highest score of ACE and catalpol was obtained. In order to verify the results of molecular docking and explore the binding ability between catalpol and ACE, the binding coefficient between catalpol and ACE at different temperatures and different concentration ratios was determined by isothermal titration calorimetry.  Figure 7 shows the isothermal titration thermal curve of the interaction between catalpol and ACE. It is clearly observed that the reaction heat of catalpol and ACE is negative, resulting in a downward peak, which indicates that the binding is an exothermic reaction. The values of *K*, ΔH, and ΔS of the fitted reaction are listed in Table 4. By comparing the value of *K*, it is judged that the binding capacity is the highest at 750 nm catalpol and 75 nm ACE at 30°C. Compared with the control group, the binding constant of 75nACE titrated by 750 nm catalpol at 25°C, 75nMACE by 750 nm at 30°, and 125nMACE by 1250 nm at 30°C was much higher than that of the control group, indicating that there was a strong binding force between catalpol and ACE.

### 3.7. ROC Curve Evaluation Results

**Figure 8:**
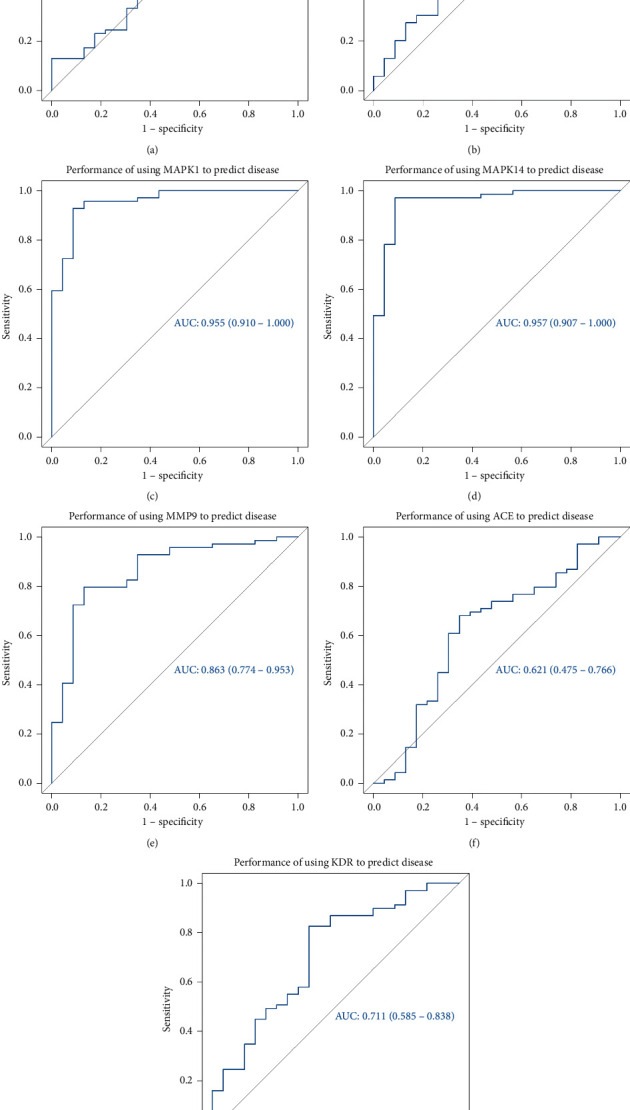
Results of ROC showed that these targets are related to stroke.

From the GEO database, we obtained 92 sets of transcriptome data. Then, we did the ROC analysis of ALB, CASP3, MAPK1, MAPK14, MMP9, ACE, and KDR targets. The results can be used to predict gene expression and achieve the role of disease prediction. The R language (version 3.6) was used for all statistical analyses. All statistical tests were bilateral, *P* < 0.05 was statistically significant. ROC results are shown in Figure 8. When the results are 0.5 < AUC < 1, it shows that the result is better than the random guess. The result has predictive value. The larger the AUC value, the stronger the predictive ability. ROC results showed that the AUC values of the key targets are all >0.5. It shows that these targets are related to stroke. The results of molecular docking are verified to some extent.

## 4. Discussion

Catalpol is one of the main active components of *Rehmannia glutinosa*, a traditional Chinese medicine for nourishing the yin and kidney, especially the content of Radix Rehmanniae [2]. Here, we apply the network pharmacology method to screen the target of catalpol prevention and treatment of stroke. Use different methods to find the target with the highest score and use the in vitro method to verify the binding force between the target and catalpol. Results showed that catalpol was involved not only in stroke but also in the skeletal system, reproductive system, infectious diseases (tuberculosis and parasites), cancer, and other diseases (see Figure 4 and Table 2), which is consistent with these studies of catalpol on multiple effects, including senile neurodegenerative diseases such as cerebral ischemia [3, 4], hypoglycemia [9, 10], diabetic encephalopathy [5], hyperlipidemia, and dementia.

The results of KEGG analysis show that the key targets of catalpol in the treatment of stroke involve multiple signal pathways related to infectious diseases such as cancer, parasites, and tuberculosis (Figure 5), which suggest catalpol mechanisms involving inflammation and cellular immunity regulation, as these diseases are mostly related to inflammatory processes and cellular immune disorders. Catalpol comes from *Rehmannia glutinosa*, which tonifies the kidney, nourishes the yin, and reduces fire (alike inflammation). Rehmannia can increase the number of T lymphocytes and enhance immunity [2]. The pathway also involves osteoblast differentiation and is related to prolactin PRL signal pathway. Catalpol can improve the proliferation and differentiation ability of osteoblast cell line MC3T3-E1 [11].

As far as the target of catalpol in the prevention and treatment of stroke is concerned, it mainly involves the following aspects.

### 4.1. Catalpol Affects the Process of Oxidative Stress, Inflammatory Injury, and Apoptosis after Ischemia

Nerve cells are very fragile and extremely sensitive to ischemia and hypoxia [12]. After cerebral ischemia, energy deficiency and metabolic disorder can induce cell apoptosis and even necrosis [13, 14]. It is very important to restore blood flow as soon as possible and save dying brain cells [15]. The results show that catalpol targets involve a large number of oxidoreductases (see Table 1), while catalpol has phenolic hydroxyl-OH group, which is prone to redox reaction and has the effect of antioxygen free radical damage, which has been confirmed in many literatures [16, 17].

PI3k-Akt signaling pathway plays an important role in cell proliferation and apoptosis [18]. Cerebral ischemia will aggravate the inflammatory reaction, further trigger a series of complex pathophysiological processes, and then lead to brain injury [19]. Catalpol can effectively relieve cerebral ischemia-reperfusion injury, reduce inflammatory reaction, and reduce the contents of IL-1 *β*, IL-6, and TNF-*α* [20]. MAPK14 is an important member of the MAPK family. It is a kind of serine/threonine protein kinase in cells. Its main function is to cause apoptosis and inflammation. Catalpol may affect PI3K-Akt-Bad [21], MAPK and IGF1, and JAK-STAT signaling pathways and inhibit inflammation and neuronal apoptosis. These reseaches give the strong evidence to support network pharmacology results that the key genes, proteins, and signal pathways affected by catalpol are involved in a variety of cell growth and development, apoptosis, energy metabolism, and inflammation (Figure 5, Table 2).

### 4.2. Catalpol Participates in the Regulation of Vascular Homeostasis and Vascular Remodeling after Cerebral Ischemia

Hemorheology and hypoxic-ischemic injury after cerebral ischemia can affect vascular homeostasis by regulating the function of vascular endothelial cells [22]. Endothelial injury and the release of inflammatory factors by inflammatory cells initiate the process of angiogenesis and participate in vascular homeostasis and vascular remodeling after cerebral ischemia [23]. The results of network pharmacology suggest that catalpol is involved in the regulation of inflammation, vascular homeostasis, and vascular remodeling after cerebral ischemia, which is closely related to F2, MMP9, VEGF/KDR, and ALB. F2 plays a role in blood homeostasis, inflammation, and wound healing [24]. NOS3 participates in the pathophysiological process of many diseases and has important functions such as regulating blood flow and relaxing blood vessels [25]. Catalpol may affect these enzymes and play a role in vascular endothelial repair. It is found that the level of MMP9 in peripheral blood of patients with cerebral ischemia is higher than that of normal controls [26]. The activation of MMP9 can increase the permeability of blood-brain barrier and aggravate the occurrence of brain edema [27]. Catalpol can significantly improve the damage of blood-nerve barrier after sciatic nerve injury and protect the integrity of neurovascular blood-brain barrier after stroke [28]. Whether catalpol treatment of stroke is related to MMP9 needs further verification. Many factors are involved in the regulation of angiogenesis and maturation after cerebral ischemia [29]. VEGF is a key factor in angiogenesis, which binds to its receptor KDR and initiates angiogenesis [30]. The research group confirmed that catalpol promotes angiogenesis and maturation after stroke, which is closely related to the upregulation of VEGF/KDR protein expression and the regulation of Jak2/Stat3 signal [15]. There are differences in serum albumin level and ALB level between different patients with ischemic stroke [31]. ALB before stroke may be one of the predictors of stroke severity. Studies have found that ALB is associated with the occurrence of stroke, and low albumin is often used as one of the indicators to predict poor prognosis after stroke [32]. It is speculated that ALB regulates cell or tissue leakage and maintains cell homeostasis and barrier function. How catalpol affects ALB will be an interesting question.

### 4.3. Catalpol Regulates Neuronal Protection and Remodeling after Cerebral Ischemia

The results of KEGG enrichment analysis (Table 2) show that MAPK1 is the key node and important target of catalpol. Other signal pathways are inextricably linked with it. MAPK1 plays a key role in synaptic plasticity, neural activity, and connectivity. Previous studies of the research group also found that catalpol can promote synaptic regeneration and axon growth. Catalpol may regulate the apoptosis of ischemic neurons and intact brain cells by activating the cascade of MAPK by MAPK1 [33]. As a neurotrophic factor, IGF1 forms a signal transduction network with other neurotrophic factors to maintain the survival and normal function of neurons.

### 4.4. ACE May Be Catalpol's Strong Target on Stroke or Other Diseases Related to Stroke

ACE (angiotensin-converting enzyme) is the main candidate gene for genetic susceptibility to cardiovascular and cerebrovascular diseases. ACE affects the process of atherosclerosis and leads to ischemic stroke [34]. Hypertension is considered to be an independent risk factor for stroke, and lower blood pressure can reduce the incidence of stroke by 30% to 40%. ACE also plays an important role in the regulation of blood pressure and is a key enzyme in the renin-angiotensin system (RAS). It can regulate blood pressure and the balance of water and electrolytes in the body [35, 36].

Our results suggest after the molecular docking of the hub protein with catalpol, the highest score is obtained as ACE. To further confirm the docking results, we also used ITC to test the binding force of ACE and catalpol, and the results showed that catalpol had a strong binding force with ACE (Figure 7, Table 4). ITC is a technique that can directly measure the binding energy of biological processes, which can be used to verify molecular docking, based on the accurate determination of the heat released or absorbed during the formation of the complex. It is an advanced technology that can measure all binding parameters at the same time. The thermodynamic properties can be obtained by fitting and analyzing the titration results with its own software.

At last, ROC curve was used to estimate the accuracy of predictions [37]. In network pharmacology, ROC can be used to evaluate the association between screened targets and diseases [38]. ROC curve results showed that the target of catalpol selected in this article is closely related to stroke (Figure 8). And further the reliability of the results is verified.

These results give us new research direction, expansion, and extension of catalpol in the prevention and treatment of diseases and its mechanism. In fact, catalpol is pleiotropy to treat diseases. But the abovementioned results suggest that the research scope of catalpol needs to be further expanded and deepened. In the future, we will identify more targets using multiple research methods in vitro and in vivo, such as cell culture and animal study, to further clarify the possible causes of catalpol pleiotropy.

## 5. Conclusion

In summary, this study uses network pharmacology and molecular docking methods to explore the targets of catalpol in the treatment of stroke. The molecular docking results show that the docking scores of ACE and catalpol were the highest. And the ITC results verify that catalpol and ACE have a strong binding force. In the ROC curve evaluation, the selected key targets have a certain degree of association with stroke disease, but still need further experimental verification, such as RT-PCR. Comprehensive bioinformatics can analyze the potential targets of disease. It can provide ideas for the research on the potential mechanism of drug therapy.

## Figures and Tables

**Table 1:** Target information and type attribution of catalpol in the treatment of stroke.

Gene name	Protein class
ACE	Hydrolase; protease
PLAU	Hydrolase; protease
BACE1	Hydrolase; protease
F2	Hydrolase; protease
REN	Hydrolase; protease
MMP9	Hydrolase; protease
VDR	Nucleic acid binding; receptor; transcription factor
NR3C2	Nucleic acid binding; receptor; transcription factor
PPARG	Nucleic acid binding; receptor; transcription factor
TGFB2	Signaling molecule
PGF	Signaling molecule
MAPK1	Kinase; transferase
MAPK14	Kinase; transferase
ACADM	Oxidoreductase; transferase
MAPKAPK2	Cytoskeletal protein; kinase; transferase
CBS	Hydrolase; isomerase; lyase
ALB	Transfer/carrier protein
ABO	Transferase
SOD2	Oxidoreductase
IGF1	None
KDR	None
NOS2	None
REG1A	None
JAK2	None
MAP2K1	None
HSD11B1	None
APRT	None

**Table 2:** Results of KEGG enrichment analysis.

	Term	Pathway	Genes
Cancer	cfa05205	Proteoglycans in cancer	MAPK1, MAP2K1, MAPK14, MMP9, IGF1, PLAU, KDR, TGFB2
	cfa05200	Pathways in cancer	MAPK1, MAP2K1, PGF, MMP9, PPARG, IGF1, NOS2, TGFB2
	cfa05216	Thyroid cancer	MAPK1, MAP2K1, PPARG
	cfa04014	Ras signaling pathway	MAPK1, MAP2K1, PGF, IGF1, KDR
	cfa04668	TNF signaling pathway	MAPK1, MAP2K1, MAPK14, MMP9

Parasitic disease	cfa05140	Leishmaniasis	MAPK1, MAPK14, JAK2, NOS2, TGFB2
	cfa05142	Chagas disease (American trypanosomiasis)	MAPK1, ACE, MAPK14, NOS2, TGFB2
	cfa05145	Toxoplasmosis	MAPK1, MAPK14, JAK2, NOS2, TGFB2

Angiogenesis remodeling	cfa04370	VEGF signaling pathway	MAPK1, MAP2K1, MAPK14, MAPKAPK2, KDR
	cfa04068	FoxO signaling pathway	MAPK1, MAP2K1, MAPK14, IGF1, SOD2, TGFB2
	cfa05152	Tuberculosis	MAPK1, VDR, MAPK14, JAK2, NOS2, TGFB2
	cfa04015	Rap1 signaling pathway	MAPK1, MAP2K1, PGF, MAPK14, IGF1, KDR
	cfa04380	Osteoclast differentiation	MAPK1, MAP2K1, MAPK14, PPARG, TGFB2
	cfa04917	Prolactin signaling pathway	MAPK1, MAP2K1, MAPK14, JAK2
	cfa04914	Progesterone-mediated oocyte maturation	MAPK1, MAP2K1, MAPK14, IGF1
	cfa04066	HIF-1 signaling pathway	MAPK1, MAP2K1, IGF1, NOS2

Inflammation, cancer	cfa04668	TNF signaling pathway	MAPK1, MAP2K1, MAPK14, MMP9
	cfa04151	PI3K-Akt signaling pathway	MAPK1, MAP2K1, PGF, IGF1, JAK2, KDR

Axonal growth nerve remodeling	cfa04722	Neurotrophin signaling pathway	MAPK1, MAP2K1, MAPK14, MAPKAPK2
	cfa04550	Signaling pathways regulating pluripotency of stem cells	MAPK1, MAP2K1, MAPK14, IGF1, JAK2

Cell growth, barrier	cfa04510	Focal adhesion	MAPK1, MAP2K1, PGF, IGF1, KDR

**Table 3:** Scores of catalpol docking with 10 hub protein receptors.

Target	Total score	Target	Total score	Target	Total score
ACE	6.9636	ALB	5.4468	F2	3.5522
MAPK14	6.4196	KDR	5.3004	IGF1	3.3508
REN	5.6157	MMP9	4.9773		
MAPK1	5.4931	PPARG	4.0449		

**Table 4:** Total thermodynamic parameters of the interaction between catalpol and ACE.

	*T* (°C)	*N*	*K* (M^−1^)	ΔS (cal/mol/deg)
A	25	0.499 ± 0.0524	(6.74 ± 1.24) × 10^7^	−2.34 × 10^6^
B	30	1.25 ± 0.107	(4.17 ± 4.22) × 10^8^	−1.28 × 10^3^
C	30	1.56 ± 0.184	(2.53 ± 4.15) × 10^8^	−2.21 × 10^3^
D	30	1.31 × 10^3^ ± 4.60 × 10^6^	0.797 ± 597	−1.89 × 10^5^

## Data Availability

The data used to support the findings of this study are available from the corresponding author upon request.
